# Fracture and embolization of a metal stent after a frozen elephant trunk procedure

**DOI:** 10.1093/icvts/ivad096

**Published:** 2023-06-13

**Authors:** Hirotaka Watanuki, Masato Tochii, Kayo Sugiyama, Katsuhiko Matsuyama

**Affiliations:** Department of Cardiac Surgery, Aichi Medical University, Nagakute, Japan; Department of Cardiac Surgery, Aichi Medical University, Nagakute, Japan; Department of Cardiac Surgery, Aichi Medical University, Nagakute, Japan; Department of Cardiac Surgery, Aichi Medical University, Nagakute, Japan

**Keywords:** Frozen elephant trunk, Stent fracture, Endovascular procedures, Aortic arch

## Abstract

The Frozenix J graft open stent graft has been available since 2014 in Japan. This stent is widely used for the frozen elephant trunk technique in many institutions, mainly for cases of acute type A aortic dissection and also for cases of a true aneurysm and chronic aortic dissection. We treated a rare case in which the metal wires of the Frozenix J graft were broken and embolized to the periphery half a year after being implanted.

## INTRODUCTION

The surgical method of total arch replacement (TAR) using the frozen elephant trunk (FET) was reported by Kato *et al.* in 1996 [1]. Then, the Frozenix J graft (Japan Lifeline, Tokyo, Japan) for the FET procedure received commercial approval in Japan in 2014 and has recently been used for total arch replacement for aortic dissection and thoracic aortic aneurysm. This type of stent graft is structured with an endoskeleton in which the stent part is fixed inside the artificial graft. We report a rare case in which the nitinol stent was fractured and embolized to the peripheral artery half a year after being implanted.

## CASE REPORT

**Figure 1:**
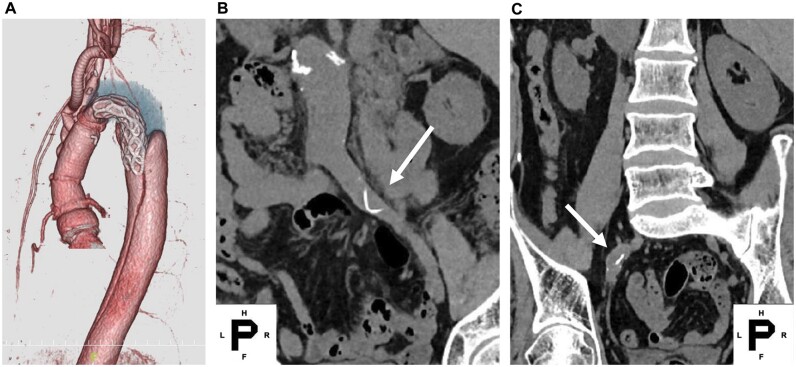
(**A**) Enhanced computed tomography scan showing the Bentall operation and total arch replacement with the flexion of the Frozenix J graft. (**B**) Computed tomography scan showing intravascular shadows of metal pieces in the right common iliac artery (arrow). (**C**) Computed tomography scan showing intravascular shadows of metal pieces in the left internal iliac artery (arrow).

**Figure 2:**
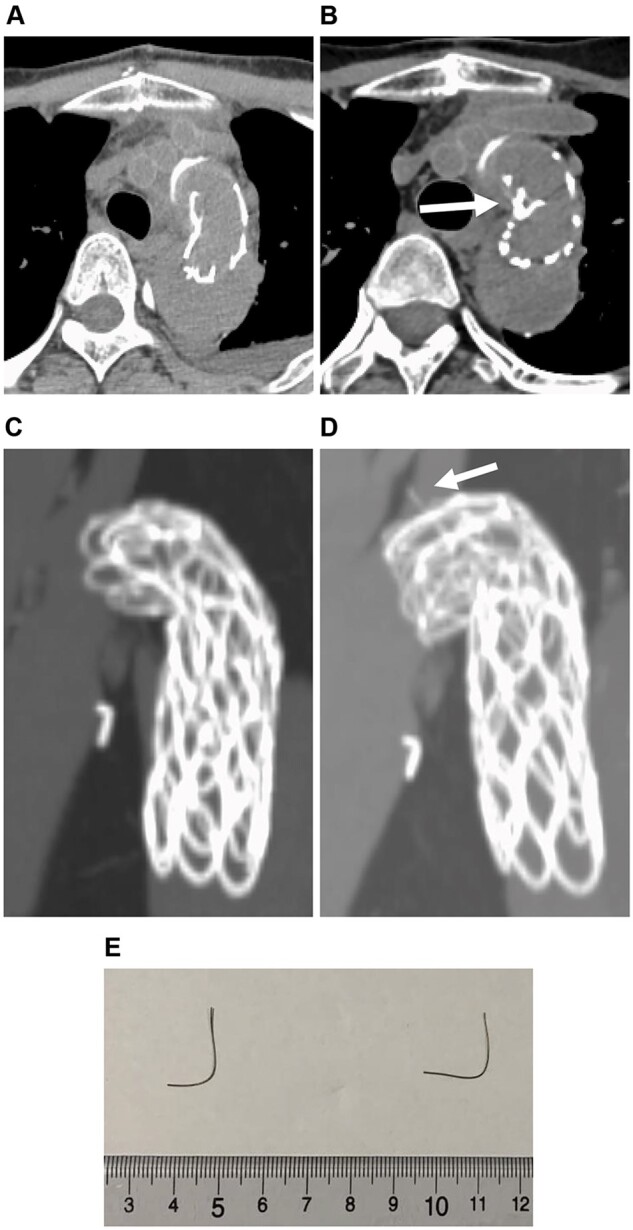
(**A, C**) Computed tomography scan immediately after total arch replacement. (**B**) Computed tomography scan showing a part of the wire that protruded into the frozen elephant trunk lumen (arrow). (**D**) Three-dimensional computed tomography scan showing a broken wire that protruded outward from the stent graft (arrow). (**E**) Each of the two picked-out metal pieces from the right common iliac artery and the left internal iliac artery.

A 46-year-old male with Marfan syndrome was referred to our hospital for back pain. He was diagnosed with acute type B aortic dissection, and an 83-mm annuloaortic ectasia with mild aortic valve regurgitation. After 1 month of conservative therapy, we performed a total arch replacement (TAR) with the open stent graft (Frozenix J graft, 23-mm diameter, 90-mm length) and a 24-mm four-branched tube graft in addition to the Bentall procedure, using a 26-mm Valsalva graft and a 23-mm mechanical valve (Fig. 1A). The stent graft size was determined to be the same size as the internal diameter of the aorta. Follow-up computed tomography (CT) 3 years after the initial operation revealed intravascular shadows of metal pieces both in the right common iliac artery (Fig. 1B) and the left internal iliac artery (Fig. 1C). CT scans performed immediately after TAR revealed no abnormal findings (Fig. 2A, 2C). However, CT scans after 6 months revealed that a part of the stent protruded into the aorta (Fig. 2B). A stent with broken continuity was confirmed (Fig. 2D) when the stent part was constructed in 3 dimensions. We explained to the patient that if the damage progressed, the stent structure may not be maintained and an embolism may recur. The patient refused to undergo additional thoracic endovascular aortic repair but agreed to have the metal pieces removed. The radiologist successfully picked out metal pieces trapped both in the right common iliac artery and the left internal iliac artery using a basket catheter without complications. The picked-out metal pieces measured approximately 2.5 cm in length (Fig. 2E). Electron microscopic analyses of the metal pieces revealed that there were some shallow abrasions and that the metal pieces were compatible with those of the Frozenix J graft. Fortunately, no further damage or embolization has been observed for 2 years.

This case report was approved by the institutional ethics committee of Aichi Medical University (2/2/2023, 2022-H046). Informed consent was obtained from the patient.

## DISCUSSION

The Frozenix J graft for the FET consists of a fabric part and a stent part constructed with a single knitted nitinol wire. The Frozenix J graft could firmly adjust to the curve of the aortic arch or a flexion of 180° or more. Furthermore, the stent is located inside the graft to prevent the former from coming into direct contact with the aorta and causing damage to the intima. This device was placed at the appropriate position simply by pulling the stent cover up under circulatory arrest without excessive depression to the stent graft during deployment. There are some reports of complications such as graft kinking and stent-induced new entry. However, stent fractures have not been reported, although more than 8 years have passed since approval from regulatory affairs was obtained. We experienced a rare case in which the broken nitinol pieces embolized to the periphery. In other stent grafts, Jacobs *et al.* reported that a stent fracture occurred in 43 (6%) of 686 endovascular abdominal aortic aneurysmal repair cases during the period; the onset varied from 1 to 48 months [2]. Heintz *et al.* reported corrosion of the nitinol wires of endovascular grafts using electron microscopic inspection in the majority of patients [3]. In this case, the wire fracture site was considered to be the lesser curvature side at the flexion of the aortic arch. Electron microscopy revealed some shallow abrasions on the surfaces of the metal pieces. The cause of the wire fractures is closely associated with anatomical factors. The flexion of the stent graft between the aortic arch and the descending aorta reached 140°, and the FET size (23 mm) was too small. A possible mechanism of wire fractures is that the wires come into contact with each other and interfere with arterial pulsation at the flexion of the stent graft, which finally causes repeated metal fatigue. We propose that this type of stent graft should be used carefully when the aortic arch has a fairly acute angle and a too-small diameter. A drawback of the Frozenix J graft is that it has a risk of peripheral embolism if the stents are damaged.

## Data Availability

The data underlying this article are available in the article.
